# Clinical Impact of Neuropostural and Neuromuscular Optimization Protocols With Radio Electric Asymmetric Conveyer (REAC) Technology in Older Adults With Femoral Fractures: An Observational Study

**DOI:** 10.7759/cureus.90221

**Published:** 2025-08-16

**Authors:** Bruna Lombardi, Margherita Imbrenda, Valtere Giovannini, Vania Fontani, Salvatore Rinaldi

**Affiliations:** 1 Research Department, Rinaldi Fontani Foundation, Firenze, ITA; 2 Physical and Rehabilitative Medicine Center, Azienda USL Toscana, Prato, ITA; 3 Physical Medicine and Rehabilitation, Casa di Cura Villa Fiorita, Prato, ITA; 4 Research Department, Rinaldi Fontani Foundation, Florence, ITA; 5 Department of Regenerative Medicine, Rinaldi Fontani Institute, Florence, ITA

**Keywords:** femoral fractures, functional dysmetria, geriatric rehabilitation, neurobiological modulation, neuromuscular optimization, neuropostural optimization, non-invasive neuromodulation, pain reduction, postural symmetry, reac technology

## Abstract

Background: Femoral fractures in the elderly often result in long-lasting disability. Standard rehabilitation may fail to address neurofunctional alterations such as functional dysmetria (FD), which limit optimal motor recovery. This study evaluates the clinical utility of Radio Electric Asymmetric Conveyer (REAC) neuromodulation protocols, Neuro Postural Optimization (NPO) and Neuro Muscular Optimization (NMO), as adjuncts to standard rehabilitation in elderly patients post femoral fracture surgery.

Methods: This observational study was conducted at Casa di Cura Villa Fiorita (59100, Prato, Italy), within a clinical-scientific collaboration program with the Rinaldi Fontani Institute. Fourteen elderly patients (mean age 81.7 years) received one NPO session, followed by 10 NMO sessions in conjunction with standard physiotherapy. A matched historical control group (n = 14) received only conventional therapy. Assessed parameters included the Barthel Index (BI), Visual Analogue Scale (VAS) for pain, FD presence, and hospital length of stay. Statistical analysis used paired/unpaired t-tests (p < 0.05).

Results: FD was completely resolved in all REAC-treated patients following the single NPO session. This neurobiological modulation likely restores subcortical and cerebellar network homeostasis, enabling symmetrical motor output. The NMO protocol supported neuromotor pattern optimization throughout recovery. Compared to controls, the REAC group achieved significantly greater improvements in BI (+47.1 vs. +38.1 points, *p *= 0.006), pain reduction (-5.2 vs. -3.6, *p *< 0.001), and shorter hospital stay (-2.2 days, *p* = 0.027). No adverse effects were reported.

Conclusions: The integration of REAC NPO and NMO protocols into conventional rehabilitation enhanced motor symmetry, pain control, and recovery speed. These findings justify further randomized trials and suggest REAC neuromodulation as a valuable, non-invasive, and scalable strategy in orthopedic rehabilitation.

## Introduction

Femoral neck fractures in older adults represent a major public health concern due to their high incidence, severe impact on autonomy, and substantial burden on healthcare systems. Despite advances in surgical techniques and physiotherapeutic approaches, many elderly patients fail to achieve full recovery, often because age-related declines in neurofunctional plasticity limit the central nervous system’s capacity for adaptive reorganization.

An underrecognized factor that can hinder optimal recovery is functional dysmetria (FD) [1,2]. Clinically, FD manifests as a persistent, measurable asymmetry in limb movement responses during standardized passive or active mobilization, not attributable to fixed structural lesions but to a reversible dysfunction in central motor control [1,2]. In conventional neurorehabilitation terminology, FD may be described as a sensorimotor control impairment [3], postural asymmetry [4,5], or interlimb coordination deficit [6]. In our theoretical framework, FD is also understood as a maladaptive neurofunctional state sometimes referred to as neuro-psycho-physical imbalance or the result of allostatic overload reflecting stress-induced dysregulation of integrated motor, postural, and autonomic systems [1,2].

Evidence from orthopedic rehabilitation research, particularly after hip fracture, has demonstrated that residual postural asymmetry and impaired interlimb coordination are associated with slower gait speed, prolonged double-support phase, reduced balance stability, increased fall risk, and delayed recovery of independence in activities of daily living [7]. For example, asymmetry in single-limb support time and variability in step velocity have been identified as key indicators of gait quality in post-hip fracture patients [8], while weight-bearing asymmetry during sit-to-stand tasks has been shown to contribute to instability and functional limitations [9].

Despite these findings, FD has not been systematically assessed in elderly orthopedic patients, and targeted interventions to correct it are absent from standard post-fracture rehabilitation protocols.

Emerging neuromodulation approaches may restore motor symmetry and enhance the effectiveness of rehabilitation. Radio Electric Asymmetric Conveyer (REAC) technology has been shown to modulate endogenous bioelectrical activity and promote neurofunctional reorganization in a non-invasive, painless, and operator-independent manner [1,10,11]. The REAC Neuro Postural Optimization (NPO) protocol has been demonstrated to correct FD through modulation of subcortical and cerebellar networks [2], while the Neuro Muscular Optimization (NMO) protocol supports coordinated neuromotor patterning and efficient muscle recruitment [10,11]. These protocols have produced positive results in diverse clinical populations [12,13], but their application in elderly patients recovering from femoral neck fracture surgery has not yet been investigated.

The aim of this study was to evaluate the clinical utility of integrating REAC NPO and NMO protocols into standard rehabilitation for elderly patients following femoral neck fracture. We hypothesized that targeted neuromodulation to correct FD could establish the neurofunctional conditions necessary to enhance autonomy, reduce pain, and accelerate hospital discharge compared to conventional rehabilitation alone.

## Materials and methods

Study design and setting

This observational study was conducted at Casa di Cura Villa Fiorita (59100, Prato, Italy), within a clinical-scientific collaboration program with the Rinaldi Fontani Institute. This study involved 14 patients (10 women, four men) with recent femoral fractures and a mean age of 81.7 years (range 94-63). Fractures were classified according to their anatomical location and pattern, based on preoperative radiographic assessment. In this cohort, the majority were intracapsular femoral neck fractures (Garden type III-IV), followed by intertrochanteric fractures (AO/OTA 31-A1 to A3) and a smaller proportion of subtrochanteric fractures. Surgical interventions were performed according to standard orthopedic indications and patient-specific factors: displaced femoral neck fractures were primarily treated with hemiarthroplasty or total hip arthroplasty, while intertrochanteric and subtrochanteric fractures underwent internal fixation using intramedullary nailing or dynamic hip screw systems. All surgeries were performed by experienced orthopedic surgeons following standard perioperative protocols.

All patients underwent a single session of REAC NPO treatment followed by 10 sessions of REAC NMO treatment, each lasting 15 minutes, administered during hospitalization. These treatments were conducted using the REAC BENE 110 medical device (ASMED, 50018 Scandicci, Italy), with the asymmetric conveyor probes (ACPs) positioned on the quadriceps. The NPO session aims to resolve FD, a neuro-psycho-motor dysfunction caused by allostatic overload [1], while the NMO protocol supports neuromuscular coordination and functional recovery [10,11].

A historical control group (n = 14) with similar age and clinical characteristics, hospitalized in the same facility and treated with conventional physiotherapy during the preceding two years, served as a comparator. Both groups were comparable in terms of the baseline Barthel Index (BI) (REAC: 36.7; control: 35.0), Visual Analogue Scale (VAS) score, age, and comorbidities. All patients provided informed consent.

Clinical parameters evaluated pre- and post-treatment included the BI for autonomy in daily activities, VAS for pain perception, and clinical assessment of FD. The length of hospital stay was also recorded. Statistical analyses included paired and unpaired Student’s t-tests, with significance set at p<0.05. Baseline characteristics of the REAC and control groups are shown in Table 1.

**Table 1:** Baseline characteristics of REAC and control groups REAC: Radio Electric Asymmetric Conveyer, VAS: Visual Analogue Scale

Parameter	REAC Group (n = 14)	Control Group (n = 14)
Age (years, mean ± SD)	81.7 ± 9.6	81.6 ± 8.9
Sex (F/M)	10/4	9/5
Initial Barthel Index	36.7 ± 7.2	35.0 ± 6.8
Initial VAS score	7.8 ± 1.1	7.6 ± 1.3
Functional dysmetria	100% present	100% present

Intervention protocol

The experimental group received one session of REAC NPO and a cycle of 10 sessions of REAC NMO. The NPO session lasted a few milliseconds and was administered with the asymmetric conveyor probe (ACP) placed on a specific point of the auricle. This protocol aims to reset neuromotor control mechanisms by modulating cerebellar and subcortical activity [2].

Subsequently, the patients underwent the NMO protocol. The NMO protocol was administered using paired ACPs placed in 10 distinct configurations to target alternating agonist and antagonist muscle groups of the lower limbs. These placements were designed to stimulate contralateral and ipsilateral neuromuscular coordination. The protocol alternated between thigh and leg muscles to optimize the reprogramming of motor patterns and postural control.

Each session involved eight minutes of stimulation per configuration, with the ACPs positioned to interact with the endogenous bioelectrical activity of the designated muscle groups. The complete cycle included 10 configurations, for example, right quadriceps with left triceps surae, left quadriceps with right triceps surae, right hamstrings with left tibialis anterior, and the corresponding contralateral combinations. This alternation aimed to engage and reorganize sensorimotor circuits involved in gait, balance, and functional mobility.

**Figure 1:**
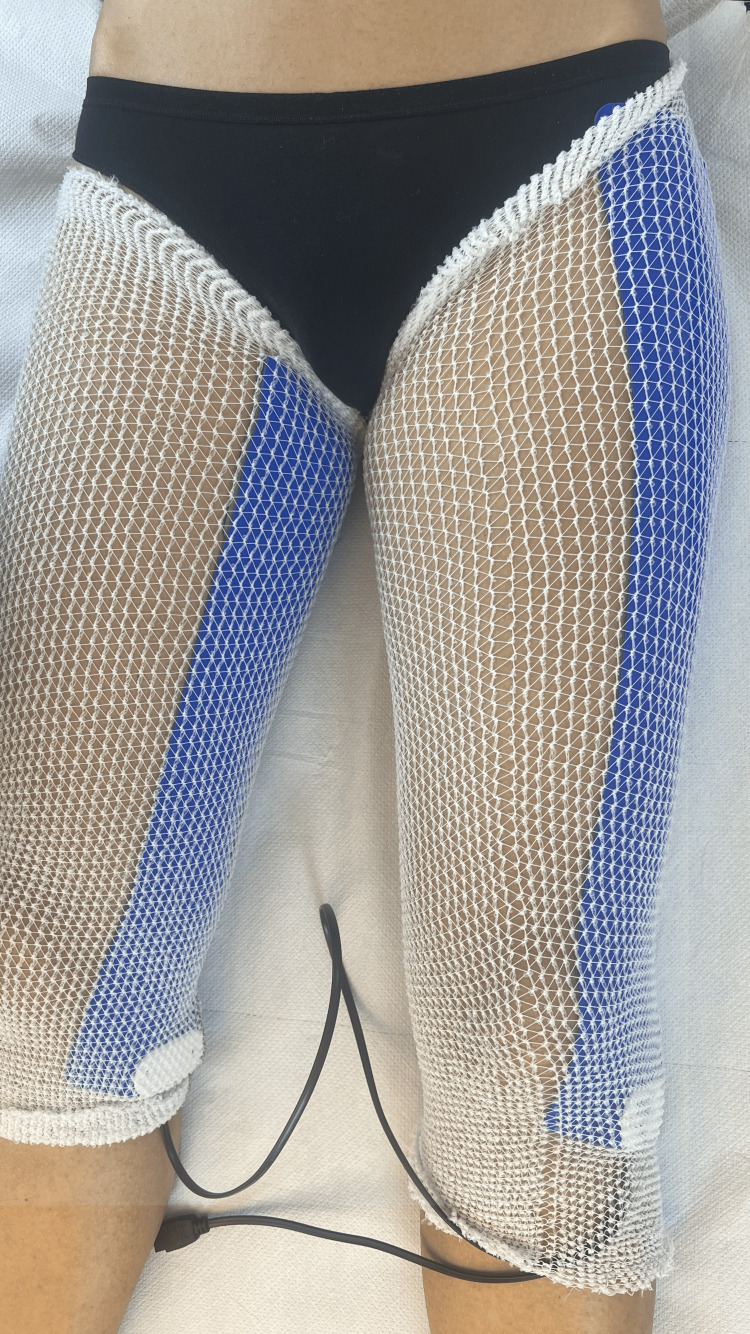
Placement of the asymmetric conveyor probes (ACPs) during one of the sessions of the REAC Neuro-Muscular Optimization (NMO) protocol. In this configuration, the ACPs target the contralateral adductor and abductor muscle groups to promote balanced neuromuscular recruitment and restore coordination between antagonistic muscles. REAC: Radio Electric Asymmetric Conveyer

The parameters for each configuration were pre-set in the REAC device and could not be altered by the operator, ensuring reproducibility. The order of muscle group targeting was consistent across all participants to maintain standardization (Figure 1).

The objective of NMO is to reinforce the symmetry of neuromuscular recruitment patterns, enabling coordinated engagement of agonist and antagonist muscle groups during movement [5,6].

All REAC treatments were administered using the BENE 110 medical device (ASMED, 50018 Scandicci, Italy). This device is designed to deliver low-intensity radioelectric emissions asymmetrically conveyed through the ACPs. Its parameters are factory-set and cannot be modified by the operator.

Physiotherapy, consisting of gait training, transfer exercises, and muscle strengthening, was conducted daily for both groups.

Outcome measures

Primary outcomes included changes in the BI [14], VAS scores [15], and FD [1].

The BI assesses the degree of independence in basic activities of daily living, ranging from 0 (total dependence) to 100 (total independence). The VAS evaluates pain on a scale from 0 (no pain) to 10 (worst imaginable pain).

FD was operationally defined as a reproducible asymmetry in quadriceps activation during the transition from supine to sitting, detected as a measurable displacement between the examiner’s bilateral thumb references placed symmetrically on the patient’s anterior thighs [1].

Patients were positioned supine on a flat, firm examination table, with the head in neutral alignment, arms relaxed alongside the body, and lower limbs extended. The physician placed both hands very lightly and symmetrically on the mid-portion of the quadriceps femoris, keeping the thumbs aligned at the same horizontal reference to serve as a “caliper” gauge. Upon a verbal cue, the patient moved from the supine to the sitting position at a comfortable, self-selected speed. FD was deemed present when, during this transition, a clear and consistent loss of alignment of the examiner’s thumbs was observed, indicating asymmetric activation of the quadriceps and, consequently, an interlimb coordination/postural asymmetry not attributable to fixed structural causes. The maneuver was repeated to verify reproducibility; FD resolution was recorded when two consecutive transitions showed maintained thumb alignment without drift.

All FD assessments were performed by two senior physiatrists (>10 years of neurofunctional evaluation experience) trained in the standardized FD procedure. Inter-rater agreement for FD detection in this study was complete, with both evaluators independently confirming all classifications, and intra-rater consistency was verified by repeated assessments yielding identical results.

Hospital length of stay was recorded.

Statistical analyses were conducted using GraphPad Prism 9 (GraphPad Software Inc., San Diego, CA). Paired t-tests compared pre- and post-treatment scores within groups, while unpaired t-tests compared the REAC and control groups. Significance was set at p<0.05.

Ethics statement

This observational study was conducted in accordance with the ethical principles outlined in the Declaration of Helsinki. All procedures involved treatments routinely used in clinical practice, and no experimental interventions or invasive methods were employed. As the study is non-interventional and observational, involving standard care supported by CE-certified medical devices (REAC BENE 110), it was reviewed and approved by the Internal Review Board (IRB) of the Rinaldi Fontani Institute (protocol number: IRB-RFI-2025-01-16). Informed consent was obtained from all patients or their legal representatives for the treatments received and for the anonymous use of clinical data.

## Results

All patients in the REAC group exhibited resolution of FD following the initial NPO session. This effect was immediate and stable throughout the hospitalization period.

**Figure 2:**
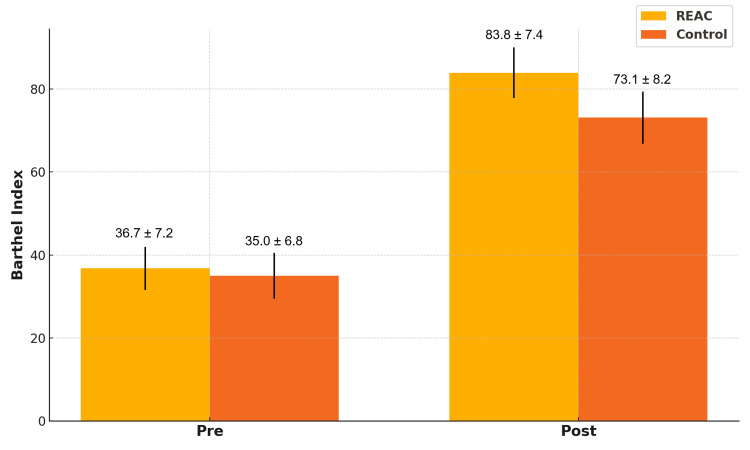
Comparison of Barthel index (pre and post) between the REAC and control groups (mean ± SD). REAC: Radio Electric Asymmetric Conveyer

In terms of functional autonomy, the BI significantly increased in both groups, but to a greater extent in the REAC group: 83.8 ± 7.4 post-treatment vs. 36.7 ± 7.2 pre-treatment, compared to the control group’s 73.1 ± 8.2 post-treatment vs. 35.0 ± 6.8 pre-treatment. The between-group difference in improvement (47.1 vs. 38.1) was statistically significant (p = 0.006), supporting a stronger recovery of autonomy in the REAC-treated patients (Figure 2).

**Figure 3:**
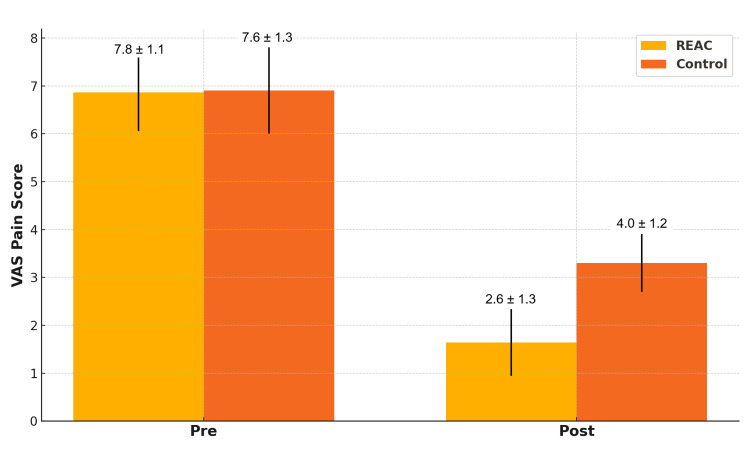
Comparison of VAS pain scores (pre and post) between the REAC and control groups (mean ± SD). REAC: Radio Electric Asymmetric Conveyer, VAS: Visual Analogue Scale

Pain levels decreased in both groups, with a greater mean reduction in the REAC group. VAS scores dropped from 7.8 ± 1.1 to 2.6 ± 1.3 in the REAC group and from 7.6 ± 1.3 to 4.0 ± 1.2 in the control group. The difference in average pain reduction (5.2 vs. 3.6) was statistically significant (p < 0.001), highlighting a more effective analgesic effect associated with REAC treatment (Figure 3).

Hospitalization duration was also affected: patients in the REAC group had a mean hospital stay of 11.1 ± 2.2 days compared to 13.3 ± 2.5 days in the control group. This difference of 2.2 days (p = 0.027) translates into significant organizational and economic advantages in rehabilitation settings.

No adverse effects were reported in any patient undergoing REAC treatment. All participants completed the intervention protocol.

## Discussion

The findings of this study suggest that integrating the REAC NPO [1,2] and NMO [10,11] protocols into conventional rehabilitation may offer a promising and safe adjunctive strategy for elderly patients recovering from femoral neck fractures. The consistent resolution of FD observed after a single NPO session [2] is consistent with previous literature demonstrating the stability of FD correction [1] and its potential role in facilitating neurofunctional reorganization across various clinical contexts [12,13,16].

In conventional neurorehabilitation terms, FD can also be described as a sensorimotor control impairment [3], postural asymmetry [4,5], or interlimb coordination deficit [6], conditions known to hinder optimal recovery after hip fracture by contributing to gait asymmetry, reduced balance stability, and delayed regaining of independence in activities of daily living [7].

While these findings are coherent with prior evidence, we acknowledge that the present study did not incorporate objective biomechanical assessments, such as gait symmetry analysis or force plate measurements, to directly confirm the functional translation of FD resolution in this population. Consequently, although the association observed between FD correction and improvements in functional autonomy, pain reduction, and reduced hospital stay is plausible, the strength of causal inference is constrained by the observational design, small sample size, and reliance on a historical control group.

The NMO protocol, aimed at neuromuscular pattern reorganization [10,11], appeared to reinforce and extend the benefits initiated by NPO [1,2], supporting more coherent motor activation and contributing to pain reduction. This potential synergy between the two protocols is reflected in the greater gains in Barthel Index scores and pain relief observed in the REAC group compared to controls. Although the reduction in hospital stay was modest in absolute terms, even small decreases in hospitalization duration may translate into clinically and economically meaningful advantages, as highlighted in cost-effectiveness analyses of post-hip fracture rehabilitation [17,18].

Compared with other neuromodulation modalities used in rehabilitation, such as transcranial magnetic stimulation (TMS) [19] or transcranial direct current stimulation (tDCS) [20], the REAC protocols offer distinct practical advantages: they are non-invasive, painless, rapid, operator-independent, require no active patient cooperation, and use preset parameters that ensure reproducibility. These features make them particularly suitable for frail elderly patients in the acute post-surgical phase. The absence of adverse effects and high tolerability observed in this and prior studies further reinforces the safety profile of REAC technology.

From a neurobiological perspective, the modulation of endogenous bioelectrical activity promoted by REAC technology [1,21,22] may contribute to restoring functional connectivity and enhancing adaptive plasticity [2,23,24]. Although not directly assessed in this study, potential mechanisms may include improved coherence of motor network signaling [2,24], reduction of maladaptive excitability [25], and facilitation of integrative processes within sensorimotor circuits, as hypothesized in prior research on stress regulation [26-28], postural control [1], and neurodegenerative conditions [16].

In summary, these preliminary results are encouraging but should be interpreted with caution in light of the methodological constraints. Future randomized controlled trials with larger sample sizes and the integration of quantitative biomechanical and neurophysiological assessments will be essential to confirm the functional impact of FD correction in elderly orthopedic populations. Such studies may also help to define the precise role of the REAC NPO and NMO protocols within multidisciplinary rehabilitation programs, potentially positioning the correction of subclinical neuromotor asymmetries as a strategic complement to task-specific training in post-fracture recovery.

## Conclusions

REAC NPO and NMO treatments provide a safe, effective, and innovative strategy to enhance rehabilitation outcomes in elderly patients with femoral fractures. The improvements in autonomy, pain control, and reduced hospital stay support the inclusion of REAC protocols in advanced rehabilitation models. Further randomized controlled trials are warranted to confirm these results and expand their applicability in broader clinical contexts.
